# Thyroid function and polycystic ovary syndrome: a Mendelian randomization study

**DOI:** 10.3389/fendo.2024.1364157

**Published:** 2024-03-22

**Authors:** Zhendan Zhao, Yuehua Gao, Xiaoqing Pei, Wenhao Wang, Ruixian Wang, Huawei Zhang

**Affiliations:** Department of Ultrasound, Shandong Provincial Hospital Affiliated to Shandong First Medical University, Jinan, Shandong, China

**Keywords:** Mendelian randomization, polycystic ovary syndrome, hyperthyroidism, hypothyroidism, free thyroxine, thyroid-stimulating hormone

## Abstract

**Background:**

Multiple evidence suggests that thyroid function is associated with polycystic ovary syndrome (PCOS), but whether thyroid function is causally related to PCOS is unclear. To investigate whether the association reflect causality, a Mendelian randomization (MR) analysis was conducted.

**Methods:**

Single nucleotide polymorphisms (SNPs) involved in this study were acquired from The ThyroidOmics Consortium and the IEU Open Genome-wide association study (GWAS) database, respectively. In forward MR analysis, we included normal free thyroxine (FT4, n=49,269), normal thyroid-stimulating hormone (TSH, n=54,288), hypothyroidism (n=53,423) and hyperthyroidism (n=51,823) as exposure. The outcome was defined as PCOS in a sample size of 16,380,318 individuals. The exposure in the reverse MR analyses was chosen as PCOS, while the outcome consisted of the four phenotypes of thyroid function. The inverse-variance weighted (IVW) method was performed as the major analysis, supplemented by sensitivity analyses.

**Results:**

The occurrence of PCOS was associated with increased risk of hyperthyroidism (IVW, OR=1.08, 95%CI=1.02-1.13, P=0.004). No evidence suggested that other phenotypes of thyroid function were related to PCOS.

**Conclusions:**

Our findings demonstrate a cause-and-effect connection between PCOS and hyperthyroidism. The study established foundation for further investigation for interaction between thyroid function and PCOS.

## 1 Introduction

Polycystic ovary syndrome (PCOS) is one of the most common diseases in women of reproductive age, with an incidence as high as 15% worldwide according to the diagnostic criteria (1). PCOS is defined by 4 clinical indicators of reproductive abnormalities, including imbalances in sex hormones, excessive secretion of androgen, persistent lack of ovulation, and the presence of polycystic ovarian morphology (2). Persistent metabolic disorders will cause a series of diseases, such as infertility and type 2 diabetes (3). Studies have revealed that the susceptibility of PCOS is influenced by interaction of gene-environment, but it is still vital to elucidate the mechanism of pathogenesis of PCOS for improving the prevention and treatment of PCOS patients (4).

Thyroid hormone is crucial for the regulation of female hypothalamic-pituitary gonadal axis, thus the correlation between thyroid function and PCOS have been extensively studied. A prospective study indicated that the incidence of PCOS is higher in patients with Hashimoto’s thyroiditis (HT) compared with people without HT (5). The study by Natalia Zeber-Lubecka et al. indicated that more mitochondrial variants were found in PCOS patients with HT compared to patients with HT only, which suggested that mitochondrial DNA genetic variants may play a crucial role in the joint occurrence of PCOS and HT (6). Another animal experiment showed that the number of follicular was lower in rats with hypothyroidism than that in control group and rats with hypothyroidism exhibited lower ovulation rate which were similar to chronic anovulation state in PCOS (7). Likewise, the changes of thyroid hormone in PCOS patients remains a subject of vivid debate. A large cohort study from Denmark confirmed that patients with PCOS had twice the risk of having hyperthyroidism compared to age-matched controls (8). However, multiple studies have shown that compared with hyperthyroidism, hypothyroidism was more common in PCOS patients (9–11). Yet there was also study which demonstrated that the risk of thyroid disease did not differ between patients with PCOS and controls (12). As observational studies are vulnerable to confounding, bias and reverse causality, it is still unclear whether the relationship between thyroid function and PCOS are causal or not.

Given the above, thyroid function is closely associated with PCOS therefore it is necessary to clarify whether the relationship involves causation. Mendelian randomization (MR) analysis, a strategy for investigating causation between different traits, is widely used to explore the casual correlation between an exposure and an outcome (9). In this study, we performed two-sample MR analysis to assess the potentially association between free thyroxine (FT4) as well as thyroid-stimulating hormone (TSH) in normal range, hypothyroidism and hyperthyroidism and PCOS. Furthermore, a reverse MR analysis was also conducted to determine the causal effect of PCOS on thyroid function.

## 2 Methods

### 2.1 Study design and data source

**Figure 1:**
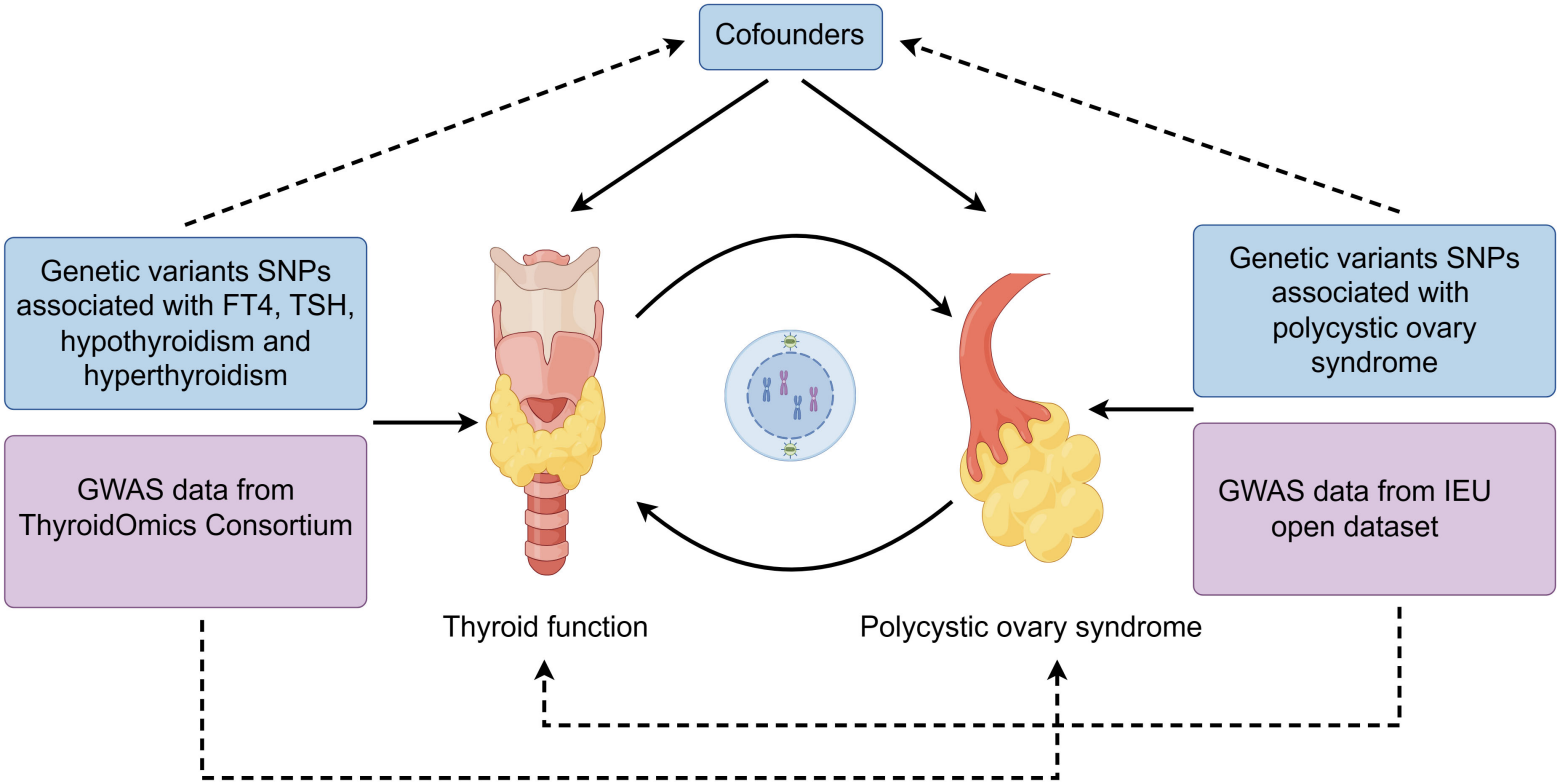
Flow chart of overall design in the present study.

The flowchart of two-sample MR analyses in this study is shown in **Figure 1**. Samples from exposure and outcome are restricted to Europeans to avoid bias caused by population stratification.

Genome-wide association study (GWAS) data for PCOS was obtained from the IEU open GWAS database (ID: finn-b-E4_POCS), which contained 642 cases and 16379676 controls. GWAS data for FT4, TSH, hypothyroidism and hyperthyroidism were derived from the ThyroidOmics Consortium database. Of these, FT4 data included 19 cohorts with 49269 subjects, TSH data included 22 independent cohorts with 54288 subjects, 53423 subjects (3440 cases) for hypothyroidism and 51823 subjects (1850 cases) for hyperthyroidism (13).

Ethical approval is not sought as the datasets in this study are publicly available.

### 2.2 Genetic variants selection criterion

We chose single-nucleotide polymorphisms (SNPs) as instrumental variables (IV) in this study.

The legitimate IVs should meet the following assumptions: (1) they are closely associated with exposures; (2) they are independent of cofounding factors; (3) they are solely related to outcomes through exposures. In forward MR analysis, the p value significance threshold of SNP was set to be 5×10^-8^. Next, linkage disequilibrium (LD) pruning was conducted to remove linked SNPs (R^2^<0.001, kb=10000). No proxy SNP was used in this MR analysis. To detect the strength of IVs, F-statistics was calculated by as β^2^_exposure_/SE^2^_exposure_ (14). SNPs with F-statistics smaller than 10 were removed. In addition, the data was harmonized by removing palindromic SNP to avoid accidental bias. In reverse MR analyses, SNPs with P<5×10^-6^ were considered significant. The following procedures was similar to the above one.

### 2.3 Statistical analysis

To estimate causal effects, inverse variance weighted method, MR Egger, weighted median, simple mode and weighted mode were used in this study. Among these, IVW method was the primary analysis. Then heterogeneity was evaluated by Cohran Q test. Mendelian Randomization Pleiotropy Residual Sum and Outlier (MRPRESSO) technique was utilized to detect and delete the SNPs with heterogeneity. Besides, we also used MR-Egger intercept and funnel plots to assess horizontal pleiotropy. Sensitivity analysis was carried out based on the leave-one-out method. Furthermore, p-values were corrected by Bonferroni adjustment (p-value/number of exposure). As a result, P 0.05/4 = 0.0125 was defined as significant. All analysis in this study were based on R version 4.2.3. The R package TwoSampleMR was used for all statistical analyses in this investigation.

## 3 Results

### 3.1 Thyroid function and PCOS: a causal link

**Figure 2:**
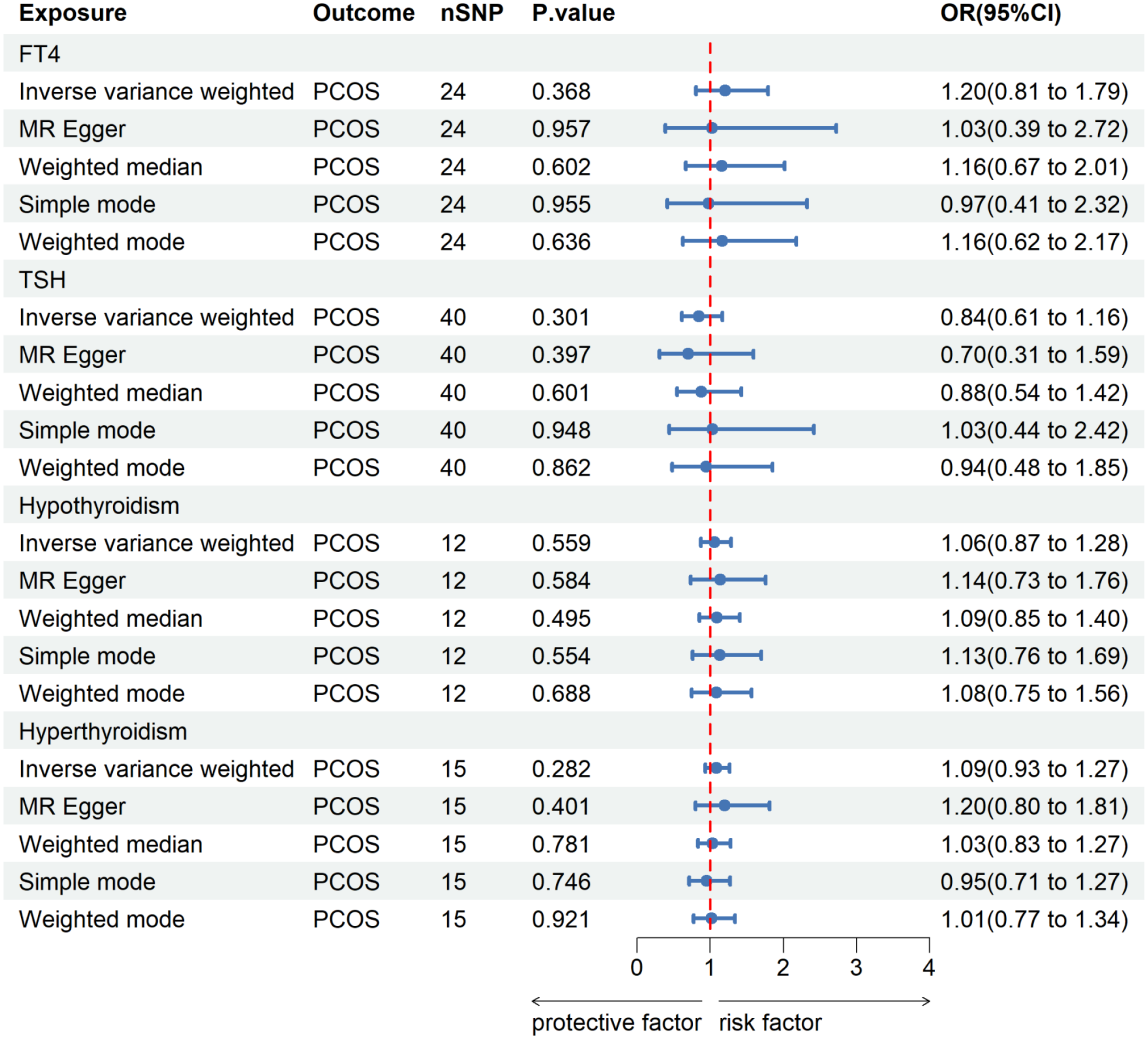
MR analysis for the causal effect of thyroid function on PCOS. IVW, inverse variance weighted; SNP, single-nucleotide polymorphism; OR, odds ratio; CI, confidence interval; PCOS, polycystic ovary syndrome; TSH, thyroid-stimulating hormone; FT4, free thyroxine.

The screening process of SNPs is shown in **Supplementary Figure 1**. After removing palindromic SNPs and outliers, we obtained 24, 40, 12 and 15 SNPs for FT4, TSH, hypothyroidism and hyperthyroidism, respectively. **Supplementary Table 1** contains all SNPs selected as instrumental variables. As shown in **Figure 2**, IVW indicated that there was no causal association between thyroid function and the risk of PCOS [for FT4: odds ratio (OR) was 1.20 at 95% confidence interval (CI) of 0.81-1.79, P=0.368; for TSH: OR was 0.84 at 95% CI of 0.61-1.16, P=0.301; for hypothyroidism: OR was 1.06 at 95% CI of 0.87-1.28, P=0.559; for hyperthyroidism: OR was 1.09 at 95% CI of 0.93-1.29, P=0.282]. Likewise, similar results were observed in MR-Egger, weighted median, simple mode and weighted mode. The Cochran’s Q test and MR Egger regression suggested no evidence of heterogeneity or horizontal pleiotropy (**Supplementary Table 2**). In addition, leave-one-out analysis and funnel plot and forest plot results are presented in **Supplementary Figure 2**.

### 3.2 PCOS and thyroid function: a causal link

**Figure 3:**
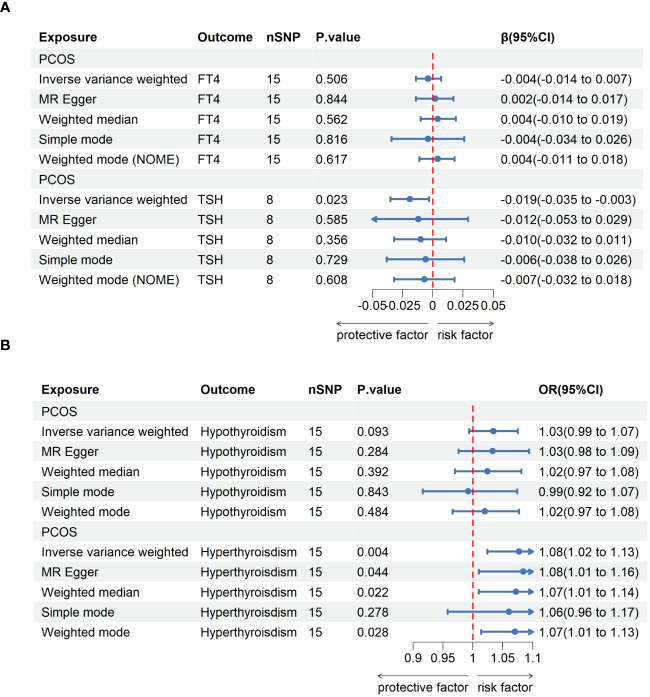
MR analyses for the causal effect of PCOS on thyroid function. **(A)**: Casual analyses of PCOS to FT4 and TSH. **(B)**: Casual analyses of PCOS to hypothyroidism and hyperthyroidism. IVW, inverse variance weighted; SNP, single-nucleotide polymorphism; OR, odds ratio; CI, confidence interval; PCOS, polycystic ovary syndrome; TSH, thyroid-stimulating hormone; FT4, free thyroxine.

In reverse MR analysis, we considered PCOS as an exposure and thyroid function as outcome. As a result of SNP filtering, 15, 8, 15 and 15 SNPs were included for FT4, TSH, hypothyroidism and hyperthyroidism, respectively, in final analysis (**Supplementary Figure 3**). Detailed information about SNP include for analysis are listed in **Supplementary Table 1**. According to **Figure 3A**, IVW findings suggested that the existence of PCOS was linked to a reduction in TSH levels (β=-0.019, 95%CI=0.035-0.003, P=0.023). Nevertheless, following the application of the Bonferroni correction, this discovery was not statistically significant. Furthermore, it was discovered that the presence of PCOS was linked to a higher likelihood of developing hyperthyroidism (IVW, OR=1.08, 95%CI=1.02-1.13, P=0.004). This association persisted after P value adjustment. The results of MR Egger and weighted median were consistent with IVW results although statistical significances were lost after Bonferroni correction (MR Egger: OR=1.08, 95%CI=1.01-1.16, P=0.044; weighted median: OR==1.07, 95%CI=1.01-1.14, P=0.022) (**Figure 3B**). No evidence of heterogeneity or horizontal pleiotropy were detected by Cochran Q test and MR Egger regression (**Supplementary Table 2**). Funnel plots and forest plots were provided in (**Supplementary Figures 4A, B)**. Furthermore, no influential study was reported by leave-one-out analysis (**Supplementary Figure S4C**).

## 4 Discussion

Our two-sample MR study provided novel evidence for causal association between thyroid function and PCOS. In our study, we found that the genetic susceptibility pf PCOS was associated with the elevated risk of hyperthyroidism, while the susceptibility of hyperthyroidism was not related to PCOS. Besides, no forward or reverse causation was observed between PCOS and FT4, TSH and hypothyroidism.

The findings of our study were in line with previous study. That study compared the percentage of thyroid disease between patients with PCOS (n=18476) and age-matched healthy controls (n=54757). As a result, the percentage of thyrotoxicosis in PCOS patients was markedly higher than that in the control group. The Cox proportional hazard model demonstrated an independent positive association between PCOS and hyperthyroidism (OR=1.7, 95%CI=1.1-2.3) (8). Additional study showed that no significant difference in thyroid disease between PCOA patients and control group. However, FT4 was elevated in PCOS group compared with control group after exclusion of patients with drug medication (12). While the effect of these drug in female hypothalamic-pituitary gonadal axis remains unknown.

However, some studies believe that a tendency towards a significant relation between PCOS and hypothyroidism. Three independent case-control studies from different countries indicated that patients with PCOS displayed a greater incidence of hypothyroidism compared to control groups (9–11). Besides, findings from another cohort study supported the established common mechanism between PCOS and autoimmune thyroiditis (AIT), which is the most common cause of hypothyroidism (15). These results are opposite to our data. Nevertheless, the study population in above results are from Pakistan, Iran, Saudi Arabia and China. So, the contradiction may be attributed to racial differences. Data from a Danish cohort study indicated that the incidence rate for thyrotoxicosis were 1.4 per 1000 patients for PCOS patients versus 0.5 per 1000 patients for patients without PCOS (8), which are consistent with our findings.

In addition, our study also revealed that PCOS had a stronger association with hyperthyroidism compared with hypothyroidism, which is in agreement with a previous study. Uma Sinha et al. found that the incidence of thyrotoxicosis was higher in PCOS patients compared to autoimmune thyroiditis (22.5% vs 2.5%) in a cross-section study in a rural population in India (16). There are two clinical manifestations of autoimmune thyroid disease (AITD): Grave’s disease (GD) and HT, both characterized by the presence of circulating antibodies in blood and lymphocytic infiltration in thyroid parenchyma (17). The clinical hallmarkers of HT and GD are hypothyroidism and thyrotoxicosis, respectively. In HT, type 1 helper (Th1) lymphocyte-mediated autoimmunity induces thyroid cell lysis and eventually leads to hypothyroidism (18). While the major mechanism of GD is hyperthyroidism caused by humoral immunity mediated by type 2 helper (Th2) cells (19).

One of the characteristics of PCOS patients is excessive androgen production. It was shown that testosterone intervention given to rats were able to increase the level of FT4 (20). Another study proved that female mice intervened by testosterone exhibited Th2-biased cytokine profiles, which suggested that androgen was capable of foster Th2-mediated-immune responses (21). This may be one of the mechanisms by which PCOS women are more likely to suffer from hyperthyroidism. In addition to that, a case-control study showed that high level of testosterone was associated with poorer performance of psychomotor speed and visual-spatial abilities in PCOS patients (22). Furthermore, it was reported that the level of testosterone was positively correlated with urine albumin-to-creatinine ratio in PCOS patients, and the follicular fluid extracted from PCOS patients with high level of serum testosterone could induce fibrotic lesion in tubular epithelial cell line (23). This reminds us that more attention should be paid to PCOS patients with higher level of testosterone to minimize complications.

Apart from autoimmune mechanism, thyroid disfunction and PCOS are closely related each other through a series of physiological metabolism. Insulin resistance is an important feature of PCOS patients (24). Study showed that insulin resistance was correlated with FT3 elevation in euthyroid subjects (25). Other study demonstrated that metformin treatment improved insulin resistance, modulated TSH level and reduced thyroid volume (26). However, the mechanism of crosstalk between thyroid function and PCOS is still unknown and needs to be further investigated.

In addition to that, our results could be interpreted from another perspective. Elena Vasyukova et al. examined the serum levels of cytokines in PCOS patients and age-matched females without PCOS. As a result, the serum concentration of CD40 in PCOS patients was significantly higher than that in controls (27). Multiple studies have shown that CD40 was significantly highly expressed in patients with hyperthyroidism and contributed to GD pathogenesis in several pathways (28, 29). On one hand, CD40 could present antigen and deliver costimulatory signals to T cells and induce the activation of T cells and ultimately enhancing the autoimmunity (30). On the other hand, overexpression of CD40 is able to activate downstream cytokines, such as IL6, which increase the production of thyroid-specific autoantibodies and promote hyperthyroidism (31). So, we speculate that overexpression of CD40 is an underlying mechanism that predispose PCOS patients to hyperthyroidism.

We have to admit that there are several limitations of our study. Firstly, due to the limitation of database, the association of other thyroid hormones such as FT3 with PCOS are not discussed in this study. Secondly, no distinction was made according to gender, so the bias caused by gender could not be avoided. Lastly, our study population was limited to European individuals, thus whether the results can be extended to other groups remains to be confirmed.

## 5 Conclusion

In conclusion, our study demonstrated that some SNPs predisposing to PCOS were associated with the development of hyperthyroidism. Further studies will be required to clarify the underlying mechanism between thyroid function and PCOS through large-scale randomized controlled trials and animal experiments.

## Data availability statement

The original contributions presented in the study are included in the article/**Supplementary Material**. Further inquiries can be directed to the corresponding author.

## Ethics statement

Ethical approval was not required for the study involving humans in accordance with the local legislation and institutional requirements. Written informed consent to participate in this study was not required from the participants or the participants' legal guardians/next of kin in accordance with the national legislation and the institutional requirements.

## Author contributions

ZZ: Conceptualization, Writing – original draft, Writing – review & editing. YG: Data curation, Writing – review & editing. XP: Data curation, Formal Analysis, Writing – review & editing. WW: Data curation, Software, Writing – review & editing. RW: Software, Visualization, Writing – review & editing. HZ: Funding acquisition, Writing – original draft, Writing – review & editing, Conceptualization.
