# Supplementary material for: Thyroid function and polycystic ovary syndrome: a Mendelian randomization study
Source: Front Endocrinol (Lausanne). 2024 Mar 22;15:1364157. doi: 10.3389/fendo.2024.1364157 (PMC10995335; doi:10.3389/fendo.2024.1364157)
Supplement: Supplementary file 1 [file DataSheet_1.docx]

Supplementary Material

## Supplementary Figures
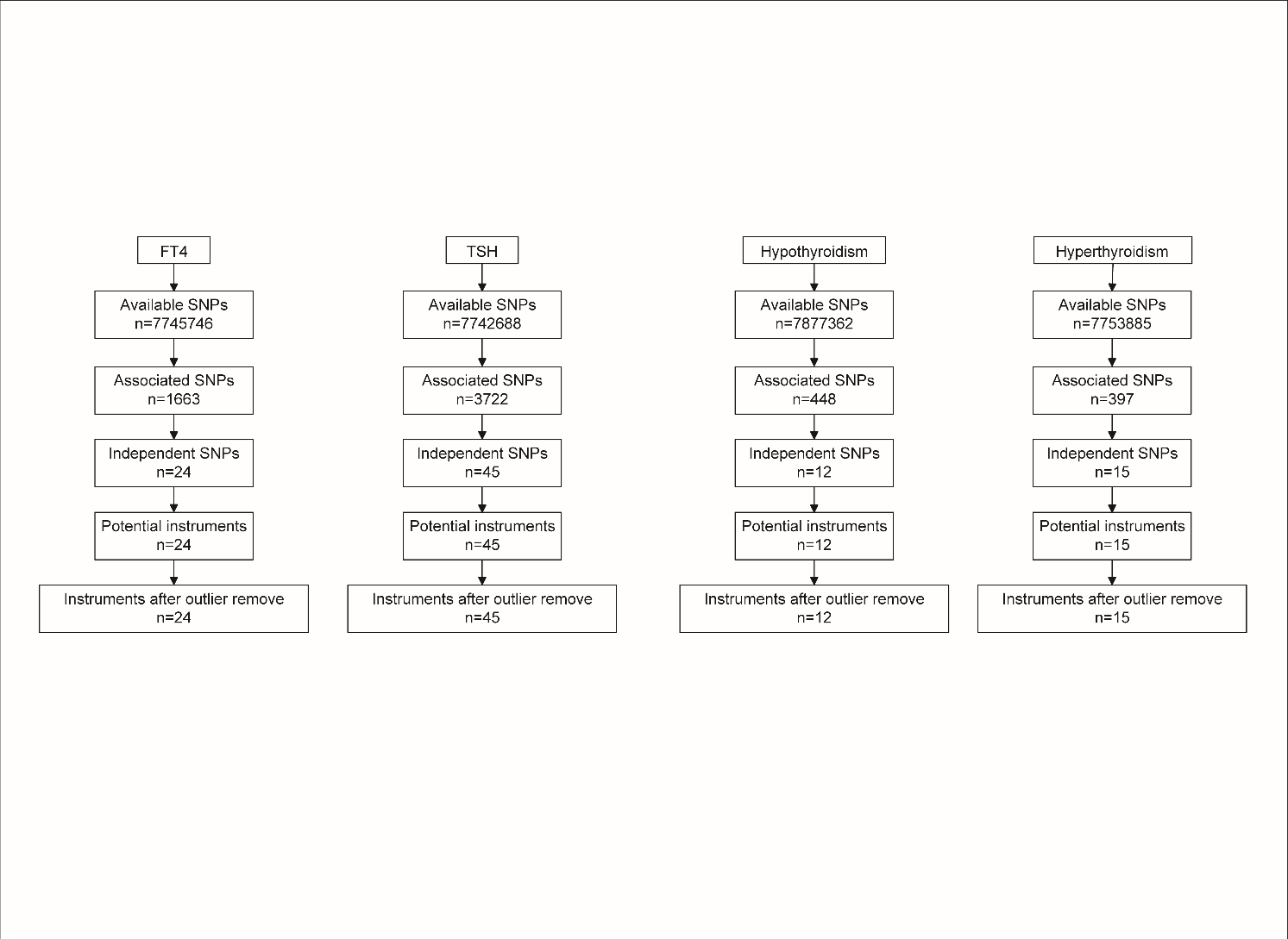


## Supplementary Figure 1. Screening process of exposure SNP in forward MR analysis.


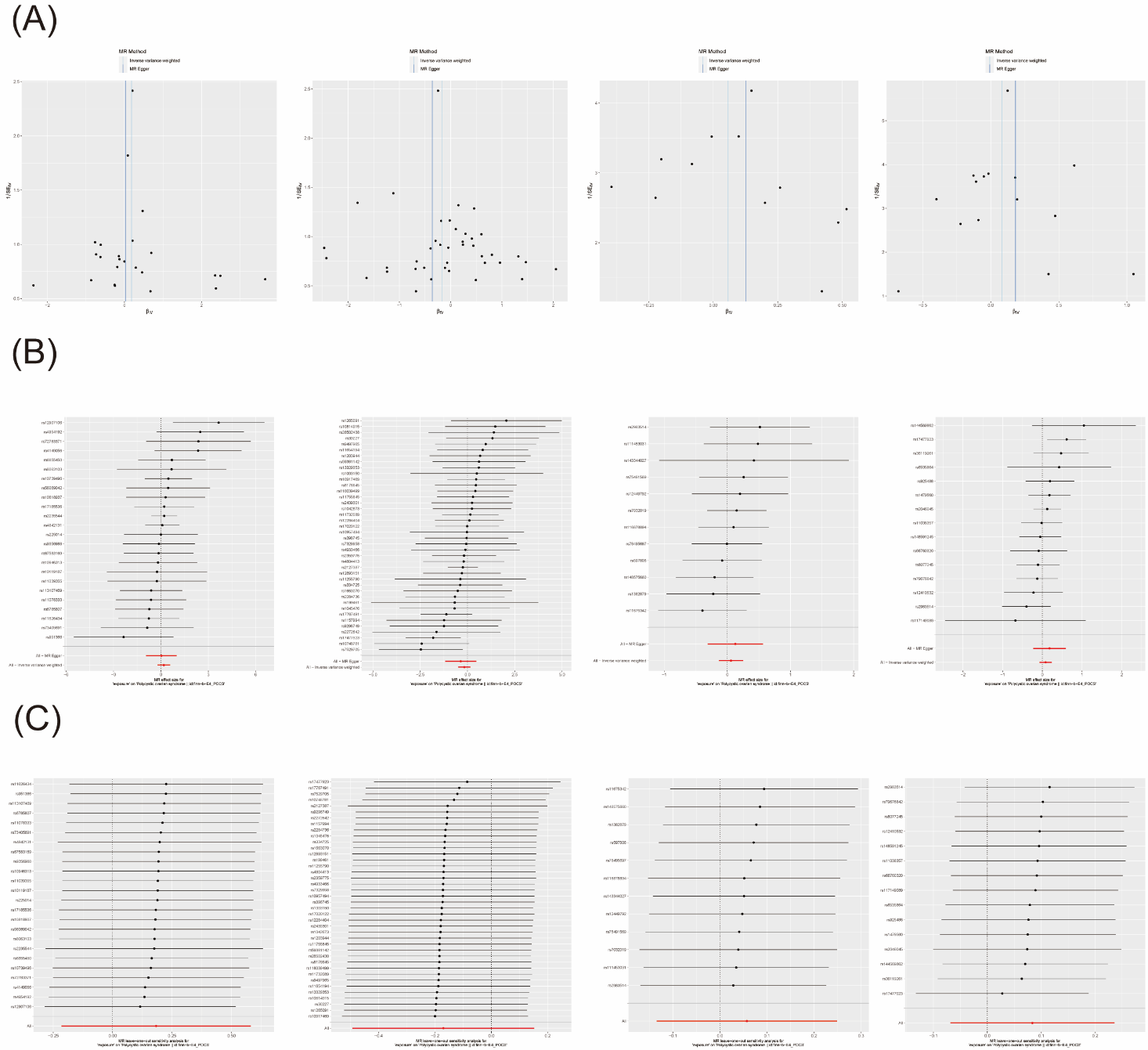
 **Supplementary Figure 2.** Funnel plots, forest plots and sensitivity analysis in forward analysis. (A) Funnel plots for causal effect of thyroid function on PCOS; (B) Forest plots for causal effect of thyroid function on PCOS; (C) Leave-one-out analysis for causal effect of thyroid function on PCOS.

**
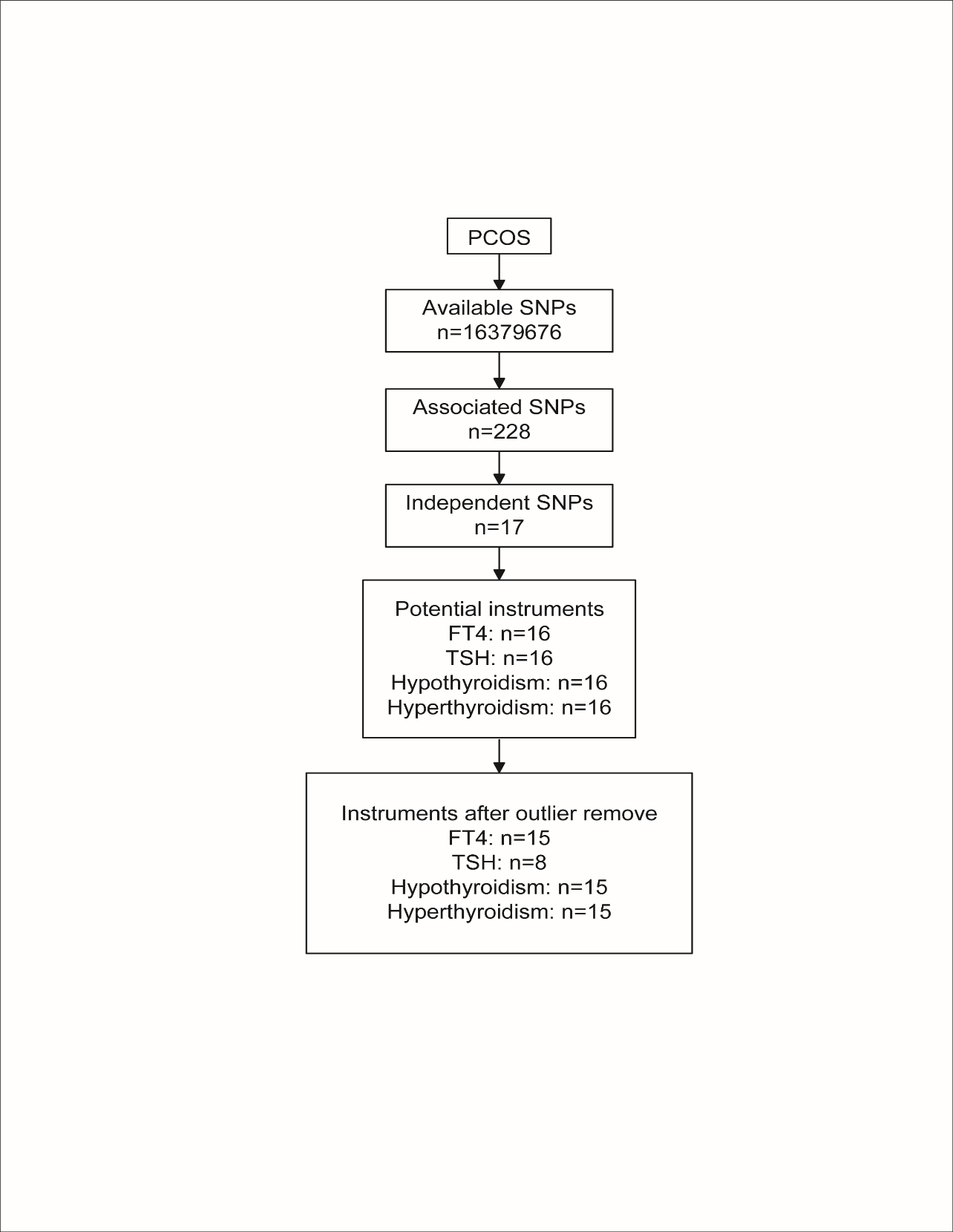
**

## Supplementary Figure 3. Screening process of exposure SNP in reverse MR analysis.


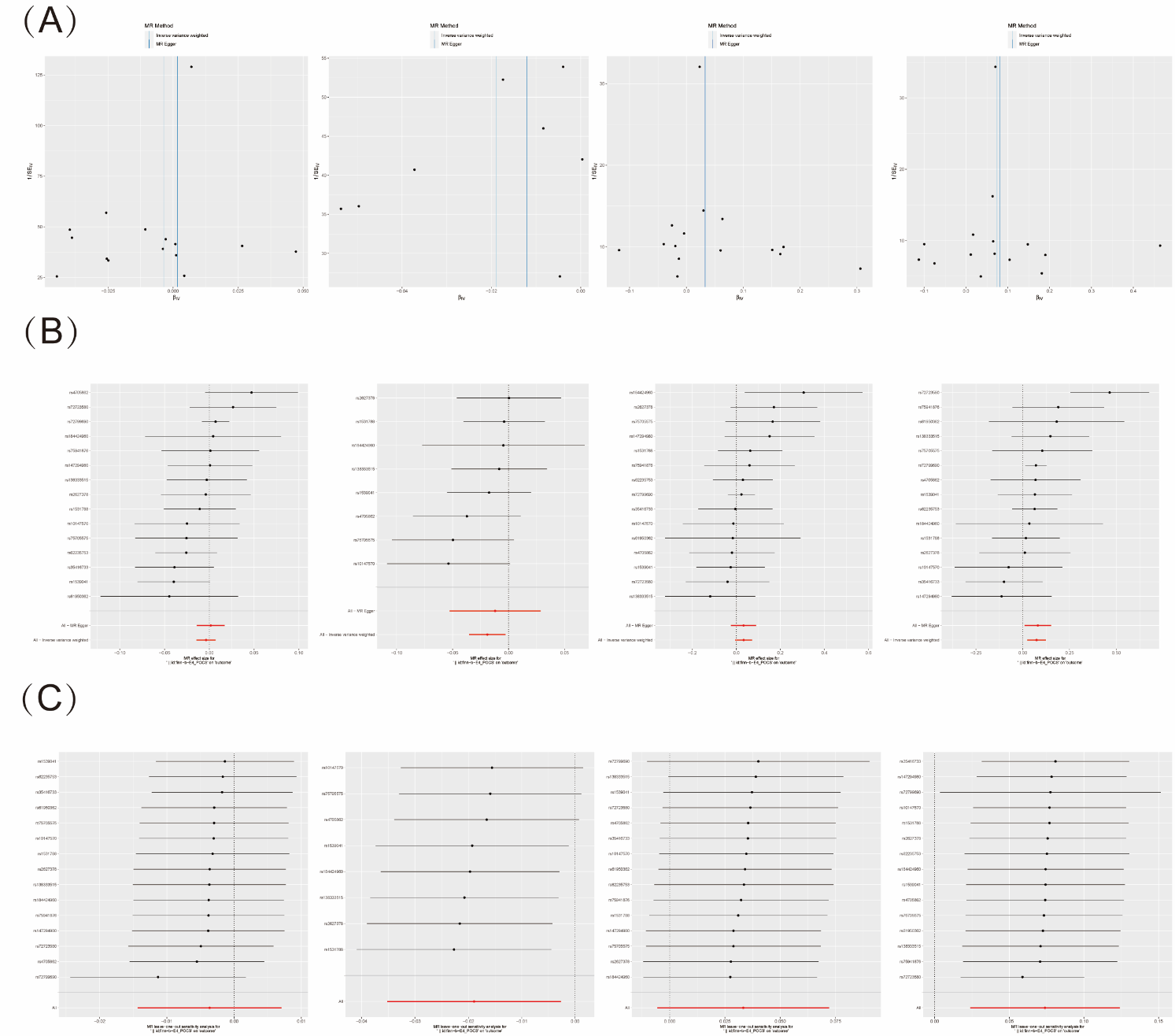


**Supplementary Figure 4.** Funnel plots, forest plots and sensitivity analysis in reverse analysis. (A) Funnel plots for causal effect of PCOS on thyroid function; (B) Forest plots for causal effect of PCOS on thyroid function; (C) Leave-one-out analysis for causal effect of PCOS on thyroid function.
